# Learning of Wheelchair Racing Propulsion Skills Over Three Weeks of Wheeling Practice on an Instrumented Ergometer in Able-Bodied Novices

**DOI:** 10.3389/fresc.2022.777085

**Published:** 2022-03-09

**Authors:** Rick de Klerk, Gabriëlle van der Jagt, Dirkjan Veeger, Lucas van der Woude, Riemer Vegter

**Affiliations:** ^1^Center for Human Movement Sciences, University Medical Center Groningen, University of Groningen, Groningen, Netherlands; ^2^Mechanical, Maritime and Materials Engineering, Delft University of Technology, Delft, Netherlands; ^3^Center for Rehabilitation, University Medical Center Groningen, Groningen, Netherlands; ^4^Peter Harrison Centre for Disability Sport, School of Sport, Exercise and Health Sciences, Loughborough University, Loughborough, United Kingdom

**Keywords:** wheelchair racing, wheelchair athletics, motor learning, propulsion technique, kinematics, physiology, kinetics

## Abstract

The acquisition of daily handrim wheelchair propulsion skill as a multi-layered phenomenon has been studied in the past. Wheelchair racing, however, is considerably different from daily handrim wheelchair propulsion in terms of propulsion technique, as well as the underlying equipment and interface. Understanding wheelchair racing skill acquisition is important from a general motor learning and skill acquisition perspective, but also from a performance and injury prevention perspective. The aim of the current lab-based study was 2-fold: to investigate the evolution of racing wheelchair propulsion skill among a sample of novices and to compare them with an experienced wheelchair racer under similar conditions. A convenience sample of 15 able-bodied novices (8 male, 7 female) completed a standardized three-week submaximal uninstructed practice protocol (3 weeks, 3 sessions per week, 3x4 min per session) in a racing wheelchair on an ergometer. Required wheeling velocity was set at 2.78 m/s (10 km/h) and a rolling friction coefficient of 0.011 (resulting in a mean target load of 21W) was used. For comparison, an experienced T54 Paralympic athlete completed one block of the same protocol. Kinetics, kinematics, and physiological data were captured. A mixed effects regression analysis was used to examine the effect of practice for the novices, while controlling for speed. All participants finished the protocol successfully. However, not all participants were able to achieve the target speed during the first few sessions. Statistically significant improvements over time were found for all outcome measures (i.e., lower metabolic strain, longer push and cycle times) with the exception of mean power and torque per push. The athlete used a significantly greater contact angle and showed “better” outcomes on most metabolic and kinetic variables. While the athlete used a semi-circular propulsion technique, most participants used a double looping over technique. Three weeks of uninstructed wheelchair racing practice significantly improved efficiency and skill among a group of novices, in line with previous studies on daily handrim wheelchair propulsion. The comparison with an experienced athlete expectedly showed that there is still a large performance (and knowledge) gap to be conquered.

## 1. Introduction

Wheelchair racing was part of the first international wheelchair sporting competition for people with disabilities in 1952 (1). Since then, wheelchair racing and racing wheelchairs have greatly evolved, the latter now consisting of a long-base three-wheel lightweight configuration with one large wheel in the front and two large rear wheels with relatively small handrims in order to reach and maintain high speeds (2). Races are organized for field and track events and include sprints, middle-long distances, and long distances, including the marathon. Athletes compete in their own class to ensure that athletes with similar impairment race against each other (3). Involvement in sports, such as wheelchair racing after rehabilitation has a positive influence on physical (4) and psychological health and well-being (1, 5). Therefore, it is important that patients with lower-limb impairments get involved in new (adapted) sports, such as wheelchair sports, during, and after rehabilitation. This requires them to learn new propulsion (and game) skills, which is especially thought to be required for wheelchair racing where the wheelchair design and interface require for different postures and propulsion technique. Although there is existing knowledge on skill acquisition during daily wheelchair propulsion (6–8), mechanisms of learning wheelchair racing are still unclear.

To become more proficient in wheelchair racing, an athlete either needs to increase the physical work capacity or become more efficient (1). Experienced wheelchair racing athletes have been studied to gain insight in their propulsion technique and corresponding mechanical efficiency. Compared with regular handrim wheelchair propulsion, athletes use a larger contact angle of approximately 180° and start at 20° past the top-dead center of the handrim (9, 10). Starting further on the handrim allows athletes to be in a more horizontal position in the racing wheelchair, reducing air resistance. Moreover, wheelchair racers use gloves as coupling is infeasible at racing speeds. During racing conditions, as segmental velocities increase, the push is performed as a stroke against the rims. Whereas, during regular handrim wheelchair propulsion one can grab the handrims, making push-pull action possible (11). To increase wheelchair racing performance, athletes need to learn this new movement, requiring different movement patterns and adaptations in both physiology and technique. Yet, little scientific research has focused on the acquisition of wheelchair racing skill thus far.

The acquisition of daily wheelchair propulsion skill has been extensively studied for regular handrim wheelchairs in wheelchair users (12) and (novice) able-bodied participants (6–8). These studies generally examined steady-state submaximal performance at low speeds, using gross mechanical efficiency as the primary outcome measure (13). Experienced participants are said to have a higher mechanical efficiency, meaning they are able to produce the same amount of external power output at a lower energetic cost. This is in line with the framework of Sparrow and Newel (14, 15) and Almåsbakk et al. (16), where cyclic movement patters are thought to emerge through the interaction of different constraints, with metabolic energy as an optimization parameter. The increase in mechanical efficiency can be due to improvements in propulsion skill and/or physiological adaptation (12). A high mechanical efficiency in wheelchair propulsion was linked to increased wheeling proficiency, expressed as greater contact angles and a decreased push frequency, which is especially beneficial as this is thought to improve mobility and reduce risk of injury (17, 18).

A better technique and higher efficiency are also beneficial to racing performance (9, 10) and could reduce injury sensitivity. However, racing and regular handrim wheelchair propulsion skill are distinct and there is no information available on the acquisition of wheelchair racing skill. One challenge specific to wheelchair racing is to maintain extreme high velocities. Smaller sized handrims help to meet the required speeds to some extent, since linear hand speed can be kept lower with smaller rim diameters which was shown to be more efficient and less straining in experienced wheelers (19). Yet, the majority of wheelchair racing performance probably still comes down to underlying coordination and skill. Like regular handrim wheelchair propulsion, wheelchair racing can be approached as a cyclical skill where motor learning can be quantified as a decrease in energy expenditure (8, 14). As such, mechanical efficiency is expected to increase, as mastering this task should result in more optimal kinetic and kinematic solutions (7, 16).

The current study focused on the initial motor learning process of three weeks of lab-based uninstructed wheelchair racing propulsion practice in inexperienced able-bodied participants on a wheelchair ergometer. More specifically, it examines the gross mechanical efficiency as the primary outcome measure for motor learning and the concomitant kinetic and kinematic solutions of the participants. Able-bodied participants were chosen as they are full novice to the task and form a relatively homogeneous group (similar age, lack of wheelchair experience, and no comorbidities), minimizing the inter-individual variation which allows to better isolate the effect of uninstructed learning on the outcomes of the motor learning process (20). Additionally, an experienced athlete performed a similar protocol to provide a reference for skilled wheelchair racing propulsion.

## 2. Materials and Methods

### 2.1. Participants

The current study used a convenience sample of 15 inexperienced able-bodied participants (7 female/8 male, 22.0 (±1.35) years old, 69.3 (±9.87) kg). The sample size was based on previous studies with a similar design in regular handrim wheelchairs (8, 21, 22). Participants were eligible for inclusion if they had no previous experience with wheelchair propulsion and no contraindications for exercise [PARQ, (23)]. Additionally, one high-level T54 middle-distance athlete, was included for comparison (male, 67 kg). All participants provided written informed consent after receiving detailed information about the study. The study was approved by the local ethical committee of the Center for Human Movement Sciences, University Medical Center Groningen, University of Groningen.

### 2.2. Study Design

**Figure 1:**
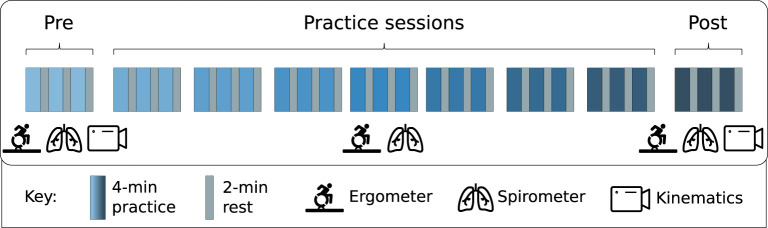
Overview of the protocol: participants were tested on 9 occasions spread over three weeks with three blocks of practice each. Data were captured during all sessions, but kinematics were only recorded during the first- and last (pre and post) session.

Participants received a total practice load of 108-min consisting of nine sessions (three sessions per week for three weeks) of 3x4 min of submaximal manual racing wheelchair exercise (Figure 1) on an instrumented wheelchair roller ergometer [Lode, Groningen, The Netherlands, (24)]. This practice load was shown to be sufficient to achieve a learning effect in regular handrim wheelchair propulsion (6, 20, 25). They received no advice on propulsion technique prior to the experiment and no feedback during the sessions, resulting in a “natural” learning process (26). Before the start of an exercise block, the sole instruction was to propel at a required speed of 2.78 m/s (10 km/h) and to hit/push the handrim with the soft hand gloves. The required velocity was based on a pilot determining a feasible, yet fast enough, speed for untrained participants. A computer screen in front of the participants provided visual feedback on the actual and target speeds (21).

### 2.3. Equipment

#### 2.3.1. Wheelchair

All tests were performed in the same experimental Amasis racing wheelchair (Wolturnes, Nibe, Denmark) with 0.71 m (28-inch) wheels and 0.38 m (15-inch) handrims on the roller ergometer. The wheelchair was not individually accommodated. Participants used soft hand gloves to push the handrim. The athlete performed in his personal racing wheelchair with 28-inch wheels and 37 cm diameter handrim. Tire pressure of the rear wheels was set at 800 kPa (8 bar) before every session.

#### 2.3.2. Physiology

Metabolic data were collected using a K5 Cardio-Pulmonary Exercise Testing (CPET) spirometer (COSMED, Rome, Italy) in breath-by-breath mode. Turbine, room air, reference gas, and delay calibrations for the spirometer were performed before each session. Heart rate was measured with a Garmin HRM Dual (Garmin International Inc, Kansas, USA) connected with the CPET. Participants were asked to rate their perceived exertion on a 6-20 Borg scale (27).

#### 2.3.3. Kinetics

Force and velocity data were collected with an Esseda (Lode BV, The Netherlands) wheelchair ergometer at 100 Hz [for a technical description see (24)]. The ergometer was calibrated to account for static and dynamic friction before each session. For a demonstration of this process see (28). A rolling friction coefficient of 0.011 was set, resulting in a theoretical power output of 21 W at the mean body weight of the novice participants in this study. The coefficient was based on eight coast-down tests (29) with two athletes at the outdoor athletics track at the Olympic Training Center Papendal. The athlete, originally part of another study, performed at a power output of 28 W.

#### 2.3.4. Kinematics

Finally, an active cluster marker was placed on the right-hand glove and tracked by an optoelectronic camera system (Optotrack, Northern Digital, Waterloo, Canada) at 100 Hz. The cluster was used to determine the location of second and fifth metacarpal (M2 and M5) during propulsion.

### 2.4. Analyses

All analyses were performed in Python [The Python Foundation, (30)] using a custom package available on the Python Package Index (31). To examine the motor learning process over time, all blocks were included and the last minute of each block was used, assuming steady-state propulsion. Finally, the mean of the three blocks per session were used for statistical analysis. Pre-processed data are available as a supplementary material file in a comma separated values (.csv) format (32).

#### 2.4.1. Physiology

Heart rate, respiratory exchange ratio (RER), and energy expenditure (EE) were obtained from the CPET system. Gross mechanical efficiency (GME) was calculated from the EE and the external power output (PO) obtained from the ergometer:


(1) $$\begin{array}{r} GME\left(\%\right)=EE*PO^{-1}*100 \end{array}$$

**Figure 2:**
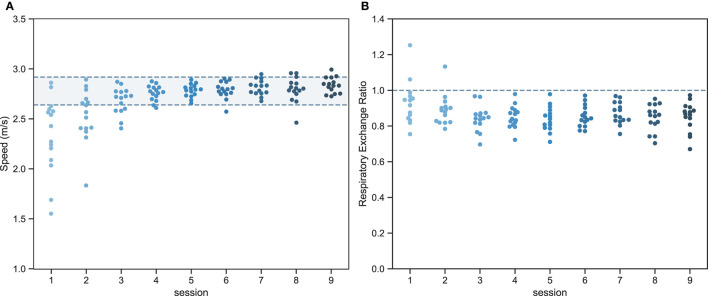
Swarmplot of the ability of individual participants to match the target speed ±5% **(A)** and the respiratory exchange ratio (RER) <1.0 **(B)** during each session (n=15).

GME for sessions where the mean RER was higher than 1.0 were discarded, which was the case for three samples (Figure 2).

#### 2.4.2. Kinetics

Kinetic data (force on the roller) from the ergometer were first filtered using a 15 Hz 4th-order zero-phase Butterworth filter. Propulsion technique variables (contact angle, push & cycle time, mean & peak torque and power per push, and work per push) were then determined based on the speed and force data from the ergometer for the left and the right side. Afterwards, the mean of the left and right side was used for further (statistical) analysis.

#### 2.4.3. Kinematics

The last fifteen s of the M2 virtual marker location were plotted for each block of the first- and last session. Three raters (GJ, RK, and PW) qualitatively rated the propulsion technique using the definitions of Boninger et al. (33): Arcing (ARC), double looping over propulsion (DLOP), semicircular (SC), and single looping over propulsion (SLOP). Participants using the ARC pattern follow the pushrim closely for a small arc during the push and recovery phase. The DLOP pattern is characterized by the hands starting above the pushrim, then following the handrim, and then going over, crossing, and going under the pushrim during the recovery phase. In the SC pattern the hand dips under the handrim in a circular or elliptic motion and in the SLOP pattern the hand passes over the handrim during the recovery phase (33). The most frequent technique among blocks was identified as the session technique. In the case of a tie, the observed technique of the last block was used, this was done for each rater individually. Finally, the most frequent technique among raters was determined and reported.

### 2.5. Statistics

#### 2.5.1. Physiology and Kinetics

A linear mixed effects analysis of the effect of time (session 1–9) was performed using R [R Core Team, (34)] and the lme4 package (35). Time and speed (without interaction term) were included in the model as fixed effects. Speed was added as not all participants were able to achieve the target velocity during the first sessions (Figure 2). Separate random intercepts and slopes were added for participants for the effect of time. The final model was defined as:


(2) $$\begin{array}{r} outcome\sim session+speed+\left(1+session|subject\right) \end{array}$$

There were no obvious deviations in the residual plots with regards to homoscedasticity or normality. P-values were obtained with a likelihood ratio test of the full model vs. a model without the effect of time. Data from the last session were compared with the athlete using a one-sample *t*-test. An alpha of 0.05 was used for all statistical tests.

#### 2.5.2. Kinematics

Fleiss' Kappa was calculated to determine the agreement between raters with regards to the propulsion technique and were interpreted based on the suggestions of Landis and Koch (36). A contingency table was produced to describe the development of propulsion technique, but no further statistical analysis was performed due to the sparsity of the data.

## 3. Results

All participants completed the experiment successfully. Yet, not all novices were able to achieve the desired velocity (±5%) during the first three sessions (Figure 2). Resultingly, speed and power output significantly increased between subsequent sessions as participants were increasingly able to achieve the target speed (Figure 2). Concomitantly, the average respiratory exchange ratio was higher than 1.0 during the first two sessions for some of the participants (2/15 in session 1 and 1/15 in session 2).

### 3.1. Physiology and Kinetics

**Figure 3:**
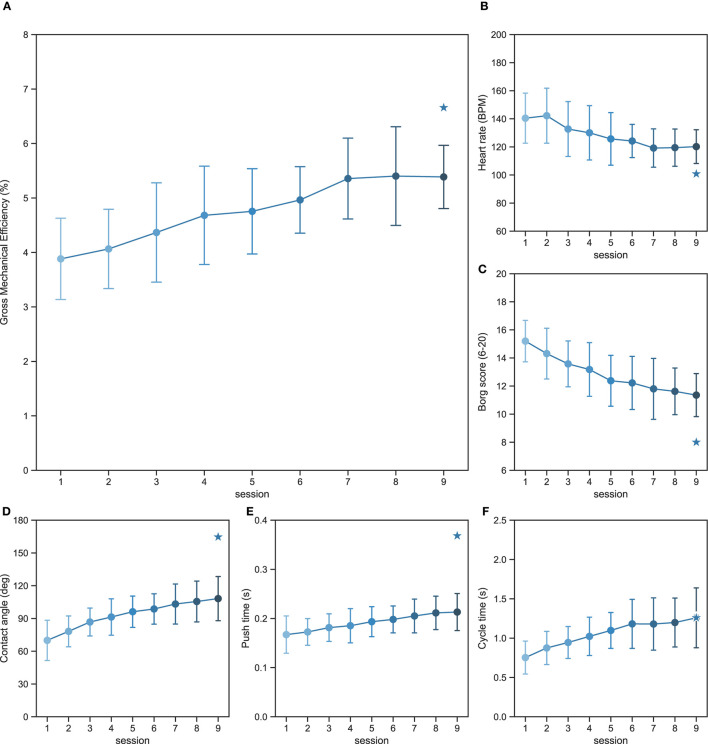
**(A–F)** Outcomes for six metabolic and kinetic parameters aggregated (mean and standard deviations, n=15) by session (⋆=athlete). All variables shown significantly changed over time. The athlete scored significantly “better” on all parameters shown, except for cycle time **(D)**.

Physiological and kinetic aggregates and statistical outcomes are displayed in Figure 3 and Table 1. A statistically significant improvement (i.e., higher GME, lower metabolic strain, higher push and cycle times) over time was found for all outcome measures with the exception of mean power and mean torque per push. Moreover, the perceived exertion also significantly lowered over time from “hard” to “fairly light.” The athlete showed significantly better outcomes (i.e., less straining) on most metabolic and kinetic variables.

**Table 1:** Outcomes: last minute of each block aggregated by session and reference data of a single wheelchair athlete with mixed effects regression and one-sample *t*-test results (n=15).

**Variable**	**Session**								**Statistics**											
	**1**	**2**	**3**	**4**	**5**	**6**	**7**	**8**	**9**	**A^***a***^**	**Speed^***c***^**	**Time^*c*^**	**χ^2^**	**p^***d***^**	**t^***e***^**	**p**
**Protocol**																
Speed	2.34 (0.39)	2.53 (0.27)	2.68 (0.15)	2.76 (0.08)	2.78 (0.08)	2.79 (0.09)	2.81 (0.08)	2.79 (0.14)	2.84 (0.08)	2.78	n.a.	2.45 (0.07)	13.987	<0.001	2.60	0.02^*f*^
Power	19.7 (4.49)	21.6 (3.55)	22.7 (3.15)	23.6 (3.2)	23.6 (3.04)	23.7 (3.03)	23.8 (2.97)	23.7 (3.20)	24.1 (2.97)	28.4	n.a.	0.43 (0.09)	13.195	<0.001	-5.50	<0.001^*f*^
**Physiological**																
RPE (6-20)	15.2 (1.8)	14.3 (2.14)	13.6 (1.85)	13.2 (2.09)	12.4 (2.03)	12.2 (1.98)	11.8 (2.23)	11.6 (1.77)	11.4 (1.60)	8.0	-2.04 (0.47)	-0.36 (0.05)	22.937	<0.001	8.20	<0.001
HR (BPM)	141 (19.5)	142 (20.7)	132 (20.2)	130 (20.5)	126 (19.8)	124 (12.9)	119 (14.3)	120 (14.1)	120 (12.8)	101	2.36 (4.95)	-3.21 (0.56)	19.128	<0.001	6.04	<0.001
EE (W)	552 (152)	543 (115)	544 (134)	522 (123)	510 (105)	482 (72.7)	451 (71.1)	445 (61.4)	450 (58.2)	426	112 (31.4)	-21.7 (3.71)	19.105	<0.001	1.52	0.06
GME (%)^*b*^	3.88 (0.78)	4.06 (0.76)	4.37 (0.94)	4.68 (0.93)	4.75 (0.81)	4.96 (0.63)	5.36 (0.77)	5.4 (0.94)	5.39 (0.60)	6.66	0.74 (0.32)	0.18 (0.83)	18.276	<0.001	-8.20	<0.001
**Kinetics (per push)**															
Contact angle (deg)	70.0 (20.4)	78.2 (15.0)	86.8 (13.5)	91.3 (17.7)	96.2 (15.7)	98.7 (14.6)	103 (18.8)	106 (19.3)	108 (20.7)	165	28.7 (4.80)	3.12 (0.84)	10.216	<0.01	-10.4	<0.001
Push time (s)	0.17 (0.04)	0.17 (0.03)	0.18 (0.03)	0.19 (0.04)	0.19 (0.03)	0.20 (0.03)	0.21 (0.04)	0.21 (0.04)	0.21 (0.04)	0.37	0.003 (0.001)	0.006 (0.002)	8.9818	<0.01	-15.4	<0.001
Cycle time (s)	0.75 (0.23)	0.88 (0.22)	0.94 (0.21)	1.02 (0.26)	1.10 (0.25)	1.18 (0.32)	1.18 (0.34)	1.2 (0.32)	1.26 (0.39)	1.26	0.32 (0.07)	0.04 (0.01)	8.8434	<0.01	0.00	0.50
Mean torque (Nm)	6.76 (2.31)	7.1 (2.39)	7.15 (2.08)	7.56 (2.44)	7.79 (2.48)	8.07 (2.92)	7.55 (1.75)	7.69 (1.85)	7.73 (1.87)	6.17			0.0242	0.87	3.22	0.03
Peak torque (Nm)	12.4 (3.85)	12.9 (3.97)	13.2 (3.92)	13.9 (4.24)	14.4 (4.58)	15.1 (4.93)	14.6 (3.76)	15.0 (4.44)	15.0 (4.10)	13.0	2.35 (0.81)	0.24 (0.10)	4.8888	0.03	1.89	0.04
Work (J)	8.59 (3.73)	10.2 (3.85)	11.3 (3.68)	12.7 (5.21)	13.7 (5.00)	14.5 (5.8)	14.2 (4.71)	14.9 (4.87)	15.3 (5.41)	18.3	6.25 (1.01)	0.49 (0.16)	7.5477	<0.01	-2.07	0.03
Mean power (W)	51.0 (23.7)	57.2 (22.8)	60.3 (19.1)	65.5 (22.1)	68.0 (22.8)	70.5 (26.0)	66.5 (16.0)	67.2 (17.2)	68.6 (17.2)	48.1			0.0768	0.78	4.61	<0.001
Peak power (W)	93.0 (40.0)	104 (38.4)	111 (36.3)	120 (38.5)	126 (42.1)	132 (44.3)	128 (34.0)	131 (40.7)	133 (37.7)	102	54.8 (7.07)	2.06 (0.85)	5.2618	0.02	3.26	<0.01

*a, athlete, single block at higher resistance; b, cases with respiratory exchange ratio (RER) < 1.0; c, unscaled estimates ± standard errors; d, p-value from likelihood ratio test; e, one-sample t-test (df=14); f, two-sided p-value; RPE, respiratory exchange ratio; HR, heart rate; BPM, beats per minute; EE, energy expenditure; GME, gross mechanical efficiency*.

### 3.2. Kinematics

**Figure 4:**
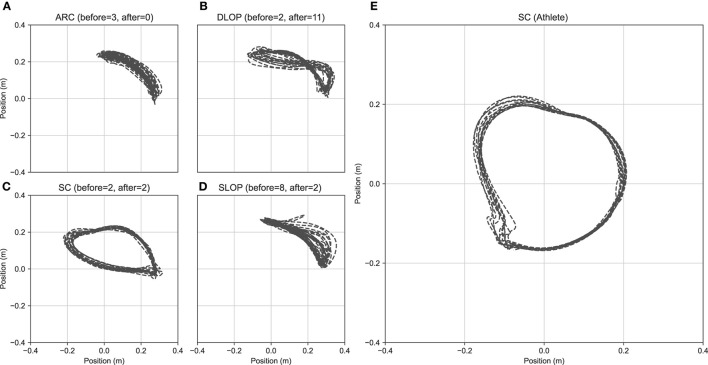
Typical kinematics examples of the last fifteen seconds of the M2 virtual marker position displayed by different participants (each subplot shows one): **(A)** arcing, **(B)** double looping over, **(C)** semicircular, **(D)** single looping over propulsion, **(E)** semicircular, athlete. All data are from the last block of the last session with the exception of panel **(A)**.

Results of the qualitative assessment of propulsion technique during the pre- and post-test are displayed in Figure 4 and Table 2. Agreement among the three raters was “substantial” during the pre-test κ=.790, p < 0.001 and “almost perfect” during the post-test κ=.813, p < 0.001. Most participants started with a SLOP (53%) technique, but the majority gravitated toward a DLOP technique in the post-test (73%). The athlete used an SC propulsion technique.

**Table 2:** Contingency table of propulsion technique during the first and last session n(%).

		**Before**				
		**ARC**	**DLOP**	**SC**	**SLOP**	**Total**
**After**	**ARC**	0 (0%)	0 (0%)	0 (0%)	0 (0%)	0 (0%)
	**DLOP**	3 (20%)	2 (13%)	0 (0%)	6 (40%)	11 (73%)
	**SC**	0 (0%)	0 (0%)	2 (13%)	0 (0%)	2 (13%)
	**SLOP**	0 (0%)	0 (0%)	0 (0%)	2 (13%)	2 (13%)
	**Total**	3 (20%)	2 (13%)	2 (13%)	8 (53%)	15 (100%)

*Arcing (ARC), double looping over propulsion (DLOP), semicircular (SC), and single looping over propulsion (SLOP)*.

## 4. Discussion

This is the first study to examine the acquisition of wheelchair racing propulsion skill within the first three weeks of practice of inexperienced able-bodied participants. In general, participants became more proficient in wheelchair propulsion in a racing wheelchair on a wheelchair ergometer, which was reflected in the successful completion of the practice bouts in terms of speed and power output, and the significant improvements in propulsion skill and corresponding reductions in metabolic cost and perceived exertion. However, the novice participants still had a significantly different propulsion technique compared to the professional athlete.

Lower heart rates and energetic cost suggest that the propulsion technique became less strenuous for the inexperienced participants over time, which is corroborated by the decrease in perceived exertion (RPE). While these lower heart rates may have been the result of improved cardiorespiratory fitness, the American College of Sports Medicine (ACSM) states that 150 min of moderate exercise, or 75 min of vigorous exercise per week are required (37). Since these requirements are not met with 108 min of exercise and as energy expenditure also decreased, the lower heart rates were more likely caused by improvement in neuromuscular coordination and thus a reduction in cardiometabolic requirements with improved coordination and skill level (20). Accordingly, gross mechanical efficiency follows an inverse pattern, increasing from 3.9 to 4.5% (+39%). However, this is relatively low compared to other studies in regular handrim wheelchair propulsion (6–8, 12, 20, 21), which is unexpected considering the relatively high power output requirements of wheelchair racing propulsion (22, 38). Experienced wheelchair racing athletes generally have a more efficient propulsion technique (10), as was the case in the current study. Yet, the results of the experienced athlete were not similar to those of experienced wheelchair racing athletes in previous studies (9, 10, 39). However, the speed and power output of the current study (2.78 m/s) were also much lower than those of previous studies (3.60–7.20 m/s), which could explain the difference in mechanical efficiency (9, 39).

Coordination of wheelchair racing propulsion is complicated due to the use of gloves, a small hand rim and a fast spinning wheel (40). Coupling happens outside of the visual field which makes it harder to start the push with the same hand velocity compared to the wheel velocity (21). As a result of practice, participants were able to increase their contact angle and decrease their push frequency, which is in line with previous studies in regular handrim wheelchairs (6–8, 12, 20, 21) and the longer-slower hypothesis as proposed by Sparrow and Newell (15). The latter states that changes in the timing of movement might be linked to reduced metabolic loads, in line with the increased muscle contraction efficiency at optimum speeds in Hill's muscle model (41). The current study adds to the body of evidence relating control parameters and metabolic expenditure. In contrast to the other parameters, mean power per push did not significantly change. However, using the same mean power on a longer push means that the participants were able to increase the amount of work delivered per push. The wheelchair athlete used an even larger contact angle, resulting in an even longer push time. Even though the athlete performed at a higher external power output, this still allowed for a lower mean and peak power per push.

Only two (13%) participants adopted a semi-circular propulsion pattern which is ubiquitous in competitive wheelchair racing. All other participants used different techniques with the majority (73%) gravitating toward a double looping over propulsion. This propulsion technique is often associated with regular handrim wheelchair propulsion (33, 42). On the other hand, athletes use a near horizontal trunk position during wheelchair racing which limits the available range of motion for the recovery pattern and forces a starting position on the handrim that is beyond top-dead center. As the current study was performed in a lab setting, where no wind or air resistance was present, there is no need to employ a more horizontal position and reduce the exposed surface area. This might have encouraged a different propulsion pattern as the task/environment constraints are different than those of actual wheelchair racing, leading to a different movement solution. However, it is still unclear whether a longer attenuation period may lead to the same kinematic solutions or that the participants are stuck in a local minimum. Finally, while pattern classification is subjective, the inter-rater agreement in this study was high. Nevertheless, some quantitative measures are available and should be further developed to provide a more robust objective method of describing propulsion patterns (42, 43).

Despite piloting beforehand, not all participants seemed able to achieve the desired velocity during the first three sessions. The able-bodied participants were complete novices, whereas regular handrim wheelchair users already have some propulsion skill that could transfer. Wheelchair racing propulsion is a relatively hard task which takes a certain amount of skill to even begin the process of mastery. To borrow terminology from the electronic-sports domain: it has a high skill floor. However, as speed was included in the mixed effects regression model, the statistical outcomes “account” for the effect of speed. The inclusion of one experienced athlete provided information about the reference technique of racing propulsion. Yet, one athlete is not representative for all wheelchair racing athletes across all disciplines. The athlete also performed at a higher external power output than the novice participants, which is known to influence propulsion technique parameters and mechanical efficiency (22, 38, 44). Finally, any potential sex-dependent differences between the athlete and 7/15 novice participants are not accounted for. These specific results should therefore be treated with care. However, for other parameters such as RPE and heart rate the differences found are even more pronounced when considering the higher power output. Finally, it must be noted that all results were obtained on an ergometer and in a small sample of able-bodied participants. The ergometer provides a more constrained, yet standardized, environment than a racing track or other training environments. Moreover, the current ergometer setup only allowed for the examination of straight-line wheelchair propulsion. Previous studies in regular handrim wheelchair propulsion, however, have not found any differences between treadmill/ergometer and overground propulsion practice (20, 45). Whether this is also the case for the more complicated wheelchair racing task is an avenue for future research.

The current study examined the effects of a uninstructed learning setup, to improve our understanding of the learning process of wheelchair racing propulsion. Yet, previous studies in daily handrim wheelchair propulsion have also examined the effects of variable practice (20) and feedback (8). Exploring those setups would be especially interesting since learning processes in sports are generally guided or supervised by trainers or coaches. The effect of feedback or variable practice could therefore provide them with valuable input. Perhaps one of the most essential parts of wheelchair racing is the coupling of the gloved hand with the handrim (40). To provide enough friction between the glove and the handrim, a medio-lateral force is required which reduces the fraction of effective force (46). Therefore, studies that specifically examine this coupling and the influence of sports equipment (i.e., rim and glove type) using 3D kinematics and kinetics are needed. Finally, since the sport is only eligible for athletes with an impairment, this seems crucial for understanding wheelchair racing. As these athletes usually already have some wheelchair experience, but might have a reduced physiological capacity or other impairments that influence the learning process. Therefore, future research should also include experienced wheelchair users that are new to wheelchair racing propulsion.

In short, the current study on motor learning processes found similar results for wheelchair racing and previous research in daily wheelchair propulsion. Similar to previous studies, participants show larger contact angles and a decreased push frequency. Using only uninstructed practice, participants increased their mechanical efficiency by 39% (1.5%-point). A comparison with an experienced athlete showed that both the propulsion pattern, and physiological and kinetic outcomes are still different. The performance gap between the participants and the experienced athletes shows that much can still be learned about the difficult task that is wheelchair racing.

## Data Availability Statement

The datasets presented in this study can be found in online repositories. The names of the repository/repositories and accession number(s) can be found at: https://dataverse.nl/dataset.xhtml?persistentId=doi: 10.34894/EBJBMF.

## Ethics Statement

The studies involving human participants were reviewed and approved by Ethische Commissie Bewegingswetenschappen, University Medical Center Groningen, Groningen, The Netherlands. The patients/participants provided their written informed consent to participate in this study.

## Author Contributions

GJ, DV, LW, and RV were involved in the conceptualization of the study, obtaining ethical approval, and writing the data management plan. GJ was responsible for the collection of the data under supervision of RK and RV. Data were pre-processed and analyzed by RK and GJ. RK drafted the final manuscript and all other authors were involved in refining the manuscript. The entire process was supervised by LW and RV.

## Funding

The preparation of this manuscript was financially supported by a grant from Samenwerkingsverband Noord-Nederland (OPSNN0109) and was co-financed by the PPP-allowance of the Top consortia for Knowledge and Innovation of the Ministry of Economic Affairs.

## Conflict of Interest

The authors declare that the research was conducted in the absence of any commercial or financial relationships that could be construed as a potential conflict of interest.

## Publisher's Note

All claims expressed in this article are solely those of the authors and do not necessarily represent those of their affiliated organizations, or those of the publisher, the editors and the reviewers. Any product that may be evaluated in this article, or claim that may be made by its manufacturer, is not guaranteed or endorsed by the publisher.
